# Association between Sleep Time and Blood Pressure in Korean Adolescents: Cross-Sectional Analysis of KNHANES VII

**DOI:** 10.3390/children8121202

**Published:** 2021-12-18

**Authors:** Suk-Won Chang, Ju-Wan Kang

**Affiliations:** 1 Department of Otorhinolaryngology, Jeju National University Hospital, Jeju 63241, Korea; swjang37111@gmail.com; 2 Department of Otorhinolaryngology, Gangnam Severance Hospital, Yonsei University College of Medicine, Seoul 06237, Korea

**Keywords:** adolescent, hypertension, diastolic, blood pressure, sleep

## Abstract

Background: Hypertension is highly related to sleep, and there have been a number of studies on sleep deprivation and the occurrence of hypertension. However, there is still insufficient research on the relationship between hypertension and various factors related to sleep. Thus, this study attempted to investigate the relationship between hypertension and sleep time-related variables in Korean adolescents. Methods: A total of 1470 adolescents (709 girls and 761 boys) between 12 and 18 years of age were enrolled through the Seventh Korea National Health and Nutrition Examination Survey (KNHANES VII). The systolic and diastolic blood pressure were measured. Sleep time-related variables such as sleep onset time, wake time, and sleep duration (weekday and weekend, each) were also investigated using a questionnaire. We performed multivariate regression analyses to determine the independent effects of the variables. Results: Systolic blood pressure was negatively correlated with the wake time (r = −0.081; *p* = 0.002) and sleep onset time (r = −0.088; *p* = 0.001) on weekends. There was a positive correlation between diastolic blood pressure and weekday sleep onset time (r = 0.158; *p* = 0.000) and weekend sleep onset time (r = 0.184; *p* = 0.000). The sleep duration on weekdays and weekends showed a negative correlation (r = −0.136; *p* = 0.000, r = −0.088; *p* = 0.001, respectively). In the multivariate linear regression analysis results, the sleep onset time on weekends was significantly correlated with elevated diastolic blood pressure. Conclusions: Delayed sleep onset time on weekends was significantly associated with increased diastolic blood pressure in Korean adolescents. Further investigation is needed to confirm the clinical significance of these findings.

## 1. Introduction

The prevalence of hypertension is increasing with economic development and is highly related to the occurrence of cardiovascular, urinary, and other diseases. There is also a growing interest in the prevention and treatment of hypertension [1,2,3]. In particular, the incidence of hypertension in adolescents has recently increased, which is a global problem leading to adult hypertension [4]. Therefore, it is important to reduce the occurrence of hypertension in adolescence through prevention and early detection such as lifestyle changes [5].

There is much ongoing research on sleep-related diseases, especially the effects of sleep deprivation on the occurrence of hypertension. The loss of sleep time has been reported as a risk factor for developing cardiovascular diseases such as hypertension or coronary heart disease [6,7,8]. In addition, in several studies on the association of hypertension incidence according to sleep time in adolescents, the deprivation of sleep time in adolescents is known as a significant risk factor for hypertension [9,10]. Several studies have been conducted on the total sleep time and the occurrence of hypertension. However, there is still insufficient research on the relationship between hypertension and sleep time-related variables such as the sleep onset time and wake time.

Therefore, this study aimed to determine the association of sleep time related variables such as the sleep onset time with the systolic and diastolic blood pressure in Korean adolescents using data from the Seventh Korea National Health and Nutrition Examination Survey (KNHANES VII) 2016–2018.

## 2. Materials and Methods

### 2.1. Study Population

We used data from the seventh Korea National Health and Nutrition Examination Survey (KNHANES VII), 2016–2018 Korea Centers for Disease Control and Prevention. The questionnaire survey included health surveys and checkups conducted at the mobile screening center. The nutrition survey was conducted by personally visiting target households. We included the data of adolescents aged between 12 and 18 years. We excluded the subjects who did not complete the questionnaire, which included variables such as sleep-related data, smoking history, and alcohol history. In addition, we excluded subjects whose blood pressure was not measured. Finally, the data of 1470 adolescents (709 girls and 761 boys) aged between 12 and 18 years were included in the analysis. We obtained informed consent from the legal guardians of all the participants. This study was approved by the Institutional Review Board of the Jeju National University Hospital.

### 2.2. Measurement of Variables

Smoking and alcohol history were also investigated using self-reported questionnaires. After the subjects rested for 10 min, four nurses from the Korea Centers for Disease Control and Prevention (ICC) specialized investigation team measured the systolic and diastolic blood pressure once in each adolescent. The blood pressure was measured thrice. Additionally, we determined the mean values of the second and third test results as the final systolic and diastolic blood pressure. The subjects’ heights and weights were also measured. The sleep onset time and wake time on weekdays were measured using a questionnaire. Sleep onset time and wake time were investigated in actual time (24-h format). However, the sleep onset time after midnight was presented by adding the actual time after 24 h in case sleep onset time. For example, 1 a.m. is marked as 25, and 2 a.m. is marked as 26. The sleep duration on weekdays was estimated from the sleep onset time and wake time in hour. The sleep onset time, wake time, and sleep duration during the weekend were also measured using the same method.

### 2.3. Statistical Analyses

We compared the two groups using the Student’s *t*-test. A correlation analysis between systolic and diastolic blood pressure and sleep time-related variables was performed. We performed multivariate linear regression analysis with diastolic blood pressure as the dependent variable. For statistical analysis, SPSS statistical software package version 17 (SPSS Inc., Chicago, IL, USA) was used. In all analyses, the *p*-value was considered to be two-tailed and the statistical significance was set at *p* < 0.05.

## 3. Results

A total of 1470 adolescents (709 girls and 761 boys) were included in the study. The mean systolic blood pressure was 111.7 (±9.8) mmHg for boys and 106.0 (±8.9) mmHg for girls; it was significantly higher in boys (*p* < 0.001). Conversely, no significant difference was observed between boys and girls in the case of diastolic blood pressure (67.6 (±7.8) vs. 67.0 (±7.8); *p* = 0.215). The sleep onset time, wake-up time, and total sleep duration were longer on weekends than on weekdays (Table 1).

Systolic blood pressure was negatively correlated with the waking up time (r = −0.081; *p* = 0.002) and the time to sleep (r = −0.088; *p* = 0.001) on weekends. There was a positive correlation between the diastolic blood pressure and weekday time to sleep (r = 0.158; *p* = 0.000) and weekend time to sleep (r = 0.184; *p* = 0.000), and the weekday sleep time (r = −0.136; *p* = 0.000) and the weekend sleep time (r = −0.088; *p* = 0.001) were negatively correlated (Table 2).

**Figure 1:**
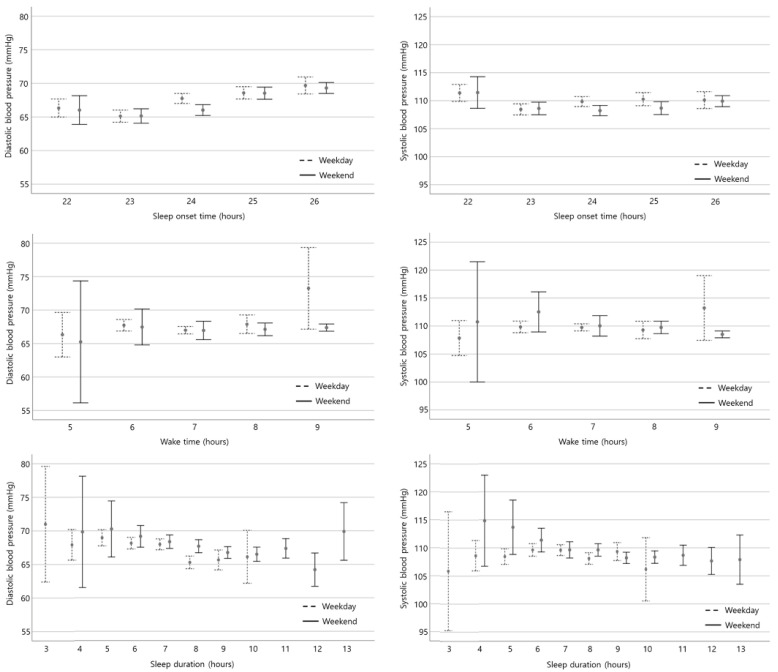
Mean (dot) and standard error (bar) according to each sleep variables. Sleep onset time and wake time are presented in actual time (24-h format). However, the sleep time after midnight was presented by adding the actual time after 24 h in case sleep onset time. For example, 1 a.m. is marked as 25, and 2 a.m. is marked as 26.

Figure 1 shows the mean and standard errors of systolic and diastolic blood pressure according to each sleep time variable. In the case of sleep onset time, it can be seen that the value of standard error was not large. However, the width of the standard error tends to be larger on the left and right sides of the sleep time measurement than in the middle area, in the case of wake time and sleep duration.

Finally, we performed a multivariate linear regression analysis. When systolic blood pressure was considered as a dependent variable, the systolic blood pressure was significantly lower in girls than in boys (B = −3.473, 95% confidence interval [CI] 2.337–4.610; *p* < 0.001), and the body weight was positively correlated with the systolic blood pressure (B = 0.302, 95% CI 0.258–0.346; *p* < 0.001). However, no variables related with the sleep time showed any significant association (data not shown).

Table 3 shows the results of the multivariate linear regression analysis when diastolic blood pressure is the dependent variable. The diastolic blood pressure increased with age (B = 0.561, 95% CI 0.267–0.854, *p* < 0.001) and was positively correlated with weight gain (B = 0.117, 95% CI 0.076–0.158, *p* < 0.001). Among the sleep related variables, only the weekend time to sleep showed a significant correlation among the factors that showed a correlation in the simple correlation analysis. In other words, as the time to sleep was delayed, the diastolic blood pressure tended to increase (B = 0.926, 95% CI 0.288–1.565; *p* < 0.001).

## 4. Discussion

This study aimed to investigate whether the sleep time-related variables such as sleep onset time, wake time, and sleep duration were related to the blood pressure in Korean adolescents. In Korean adolescents, we confirmed a significant association between the sleep onset time on weekends and diastolic blood pressure. However, other variables related to the sleep time were not correlated with the systolic and diastolic blood pressure. In addition, the sleep duration, which is known to be related to elevated blood pressure in a previous study [9,10], did not show any significant association with blood pressure in our study. Additionally, we showed that the increase in body weight was significantly correlated with the increase in systolic and diastolic blood pressure and the increase in diastolic blood pressure was significantly associated with an increase in age.

Several studies have reported a strong association between sleep deprivation and hypertension. Hwangbo et al. reported that a sleep duration of less than 6 h in Korean adults over 19 years had a significant relationship with the occurrence of hypertension. Notably, they showed that catch-up sleep on weekends significantly reduced the incidence of hypertension [11]. Moreover, one study reported that a short sleep duration was associated with hypertension in adolescents [12]. There have been several studies on the association between catch-up sleep on weekends and hypertension. One study reported that catch-up sleep on weekends was significantly reduced in hypertensive children by 40 min compared with the control group [13]. However, other studies have reported that a lack of weekend catch-up sleep was not correlated with hypertension but raised the risk of metabolic derangements such as type 2 diabetes and hypercholesterolemia [14]. As such, it is thought that there may be an association between sleep supplementation on the weekend and the occurrence of hypertension; however, there are insufficient studies to accept it as a consistent opinion. Therefore, additional prospective studies are needed on the association between hypertension occurrence and sleep time-related variables such as weekend catch-up sleep and sleep onset time.

However, there is still a lack of study on the effects of sleep or wake-up time on blood pressure. In this study, the sleep onset time, wake-up time, and total sleep duration were extended on weekends than on weekdays in Korean adolescents. It was observed that sleep is usually delayed with an early wake-up time in adolescents, and a short sleep duration due to schoolwork during weekdays; they compensate for this lack of sleep on the weekends. Another study of high school students in Korea reported that the average weekday sleep time was 5 h and 42 min (SD, 1 h). This result shows that Korean adolescents have a short weekday sleep time compared to other countries: 8.4 h in Spain, 7.8 h in India, and 8.04 h in Germany [15].

As of yet, we do not know the exact biological mechanism underlying the association between a late sleep onset time on weekends and an increased diastolic blood pressure in adolescents. However, we postulate that changes in the sleep patterns on weekends (delayed sleep onset time and late wake time) might be related to blood pressure. A preceding study showed that irregular changes in the sleep time on weekdays and weekends in adolescence are associated with brain function and circadian misalignments. These factors might be related to substance abuse problems and depression [16]. Similarly, other studies have suggested that weekday and weekend sleep time inconsistencies can affect brain function, even though weekend catch-up sleep can help recover from sleep deprivation during the weekday [17]. We can see that Korean adolescents compensate for the lack of weekday sleep on weekends. However, they go to sleep later on weekends than on weekdays. It is presumed that sleeping late on the weekends makes up for the lack of play or study time during the weekdays. We postulated that the changes in the sleep patterns on weekends (delayed sleep onset time and late wake time) might be related to an elevated diastolic blood pressure. In addition, further study is needed on what clinical significance the difference in the width of the standard error of systolic blood pressure and diastolic blood pressure measurements according to each sleep time variable.

Several studies have shown the relationship between weight gain and blood pressure in adolescents. There was a significant association between the body mass index (BMI) and blood pressure in adolescents; additionally, it has been reported that the systolic and diastolic blood pressure increased with weight gain in overweight adolescents [18]. Moreover, a study reported that weight loss was a critical factor in preventing hypertension in obese adolescents [19]. As lifestyle modifications such as weight loss are essential when adolescents with hypertension are overweight or obese, weight and blood pressure also have a significant association, as shown in the results of this study [20].

The strength of this study is that the results are reliable because we enrolled many participants compared to the study objectively measured using an actigraph. Another strength of this study is that, unlike other studies, it was possible to identify detailed factors for the occurrence of hypertension in adolescents by dividing the total sleep duration, sleep onset time, and wake-up time on weekdays and weekends. However, this study had several limitations. First, it is more likely to involve subjectivity in measuring the sleep time than a study using an actigraph. Second, in the results of this study, the diastolic blood pressure was significantly associated with the sleep onset time on weekends but not with the time to sleep on a weekday. Third, since this study is a cross-sectional analysis, it was impossible to explain the mechanism of the significant association between the sleep onset time on weekends and diastolic blood pressure. Therefore, additional prospective research is needed to clarify the mechanism of the results of this study.

## 5. Conclusions

In this study, we confirmed that a delayed sleep onset time on weekends was significantly correlated with increased diastolic blood pressure in Korean adolescents. This study showed that a late sleep onset time might be a significant risk factor for hypertension in adolescents. Further study is needed on the mechanism underlying the relationship between the sleep onset time to sleep and hypertension in adolescents.

## Figures and Tables

**Table 1:** Characteristics and clinical parameters of the study population.

Variables		Total (*n* = 1470)				
		Range	Mean (±SD)	Boy (*n* = 761)	Girl (*n* = 709)	*p* ^#^
Systolic BP (mmHg)		81–156	108.9(±9.8)	111.7 (±8.9)	106.0 (±8.9)	<0.001
Diastolic BP (mmHg)		35–94	67.3 (±8.4)	67.6 (±7.8)	67.0 (±7.8)	0.215
Height (cm)		114–189	164.7 (±8.7)	169.1 (±8.6)	160.0 (±5.9)	0.001
Weight (kg)		28–111	58.2 (±13.4)	62.4 (±14.4)	53.7 (±10.4)	0.001
Week day	Sleep onset time *	22–26	23.9 (±1.2)	23.8 (±1.1)	24.0 (±1.2)	0.001
	Wake time *	5–9	6.9 (±0.6)	6.9 (±0.6)	6.8 (±0.6)	0.001
	Sleep duration ^$^	3–10	6.9 (±1.3)	7.1 (±9.8)	6.7 (±1.4)	0.001
Weekend	Sleep onset time *	22–26	24.5 (±1.2)	24.4 (±1.2)	24.6 (±1.2)	0.001
	Wake time *	5–9	8.6 (±0.8)	8.5 (±0.8)	8.7 (±0.7)	0.001
	Sleep duration ^$^	4–14	8.8 (±1.6)	8.7 (±1.6)	9.0 (±1.6)	0.001

Abbreviations: BP, Blood pressure. * Sleep onset time and wake time are presented in actual time (24-h format). However, the sleep time after midnight was presented by adding the actual time after 24 h in case sleep onset time. For example, 1 a.m. is marked as 25, and 2 a.m. is marked as 26. ^$^ Duration of sleep is presented in hours. ^#^ Mean value of two groups were analyzed using the Student’s *t*-test.

**Table 2:** Correlation analysis of sleep-related factor with systolic and diastolic blood pressure.

		Systolic BP		Diastolic BP	
Variables		Correlation Coefficient	*p*-Value	Correlation Coefficient	*p*-Value
Weekday	Sleep onset time	0.013	0.631	**0.158**	**<0.001**
	Wake time	0.002	0.953	0.009	0.721
	Sleep duration	−0.009	0.741	**−0.136**	**<0.001**
Weekend	Sleep onset time	0.023	0.371	**0.184**	**<0.001**
	Wake time	**−0.081**	**0.002**	0.015	0.567
	Sleep duration	**−0.088**	**0.001**	**−0.088**	**0.001**

Abbreviations: BP, Blood pressure. Bold number indicates statistical significance (*p* < 0.05).

**Table 3:** The multivariate linear regression analysis when diastolic blood pressure is considered as the dependent variable.

Variables	Coefficient (95% Confidence Interval)	*p*-Value
Sex	0.516 (−0.540–1.572)	0.338
Age	**0.561 (0.267–0.854)**	**<0.001**
Alcohol history	−0.106 (−1.181–0.969)	0.847
Smoking history	0.853 (−0.515–2.222)	0.222
Height	0.007 (−0.066–0.081)	0.850
Weight	**0.117 (0.076–0.158)**	**<0.001**
Weekday sleep onset time	−0.197 (−3.200–2.806)	0.898
Weekday wake time	0.614 (−2.264–3.49)	0.676
Weekday sleep duration	0.087 (−2.738–2.912)	0.952
Weekend sleep onset time	**0.926 (0.288–1.565)**	**0.004**
Weekend wake time	−0.484 (−1.321–0.353)	0.257
Weekend sleep duration	0.167 (−0.247–0.582)	0.429

Bold number indicates statistical significance (*p* < 0.05). Sleep onset time and wake time are presented in actual time (24-h format). However, the sleep time after midnight was presented by adding the actual time after 24 h in case sleep onset time. For example, 1 a.m. is marked as 25, and 2 a.m. is marked as 26. Duration of sleep is presented in hours.

## Data Availability

All available data generated or analyzed during this study are included in this published article. Other raw data are not available due to the regulation of data sharing in the Republic of Korea.
